# Supplementary material for: Multi‐dataset identification of innovative feature genes and molecular mechanisms in keratoconus
Source: J Cell Mol Med. 2024 Sep 19;28(18):e70079. doi: 10.1111/jcmm.70079 (PMC11412914; doi:10.1111/jcmm.70079)
Supplement: Supplementary file 2 — Material S2. [file JCMM-28-e70079-s002.zip › 7.GSEA analysis/5.RNF217-AS1/KEGG_CELL_ADHESION_MOLECULES_CAMS.html]

Details for gene set KEGG\_CELL\_ADHESION\_MOLECULES\_CAMS[GSEA]

|  || Dataset | RNF217-AS1.group.cls#h\_versus\_l |
| Phenotype | group.cls#h\_versus\_l |
| Upregulated in class | l |
| GeneSet | KEGG\_CELL\_ADHESION\_MOLECULES\_CAMS |
| Enrichment Score (ES) | -0.4728486 |
| Normalized Enrichment Score (NES) | -1.5136037 |
| Nominal p-value | 0.01 |
| FDR q-value | 0.33385056 |
| FWER p-Value | 0.744 |
Table: GSEA Results Summary

  

Fig 1: Enrichment plot: KEGG\_CELL\_ADHESION\_MOLECULES\_CAMS      
 Profile of the Running ES Score & Positions of GeneSet Members on the Rank Ordered List

  

| SYMBOL | TITLE | RANK IN GENE LIST | RANK METRIC SCORE | RUNNING ES | CORE ENRICHMENT || 1 | CD86 | na | 733 | 0.481 | 0.0024 | No |
| 2 | OCLN | na | 1858 | 0.361 | -0.0123 | No |
| 3 | CLDN22 | na | 2027 | 0.348 | -0.0001 | No |
| 4 | CLDN6 | na | 4136 | 0.248 | -0.0489 | No |
| 5 | SELPLG | na | 4892 | 0.220 | -0.0599 | No |
| 6 | CLDN16 | na | 5478 | 0.200 | -0.0670 | No |
| 7 | CD99 | na | 6440 | 0.170 | -0.0864 | No |
| 8 | CNTN2 | na | 6890 | 0.156 | -0.0917 | No |
| 9 | MPZ | na | 7041 | 0.152 | -0.0886 | No |
| 10 | CD4 | na | 7120 | 0.150 | -0.0835 | No |
| 11 | ITGA9 | na | 7138 | 0.149 | -0.0767 | No |
| 12 | PTPRF | na | 7503 | 0.140 | -0.0804 | No |
| 13 | ITGAM | na | 7592 | 0.138 | -0.0762 | No |
| 14 | ITGB7 | na | 8059 | 0.126 | -0.0834 | No |
| 15 | SDC2 | na | 8258 | 0.122 | -0.0832 | No |
| 16 | CDH3 | na | 8633 | 0.113 | -0.0884 | No |
| 17 | CD28 | na | 9337 | 0.098 | -0.1039 | No |
| 18 | PDCD1LG2 | na | 9598 | 0.093 | -0.1069 | No |
| 19 | PDCD1 | na | 9790 | 0.090 | -0.1080 | No |
| 20 | CD80 | na | 10316 | 0.081 | -0.1192 | No |
| 21 | CLDN7 | na | 10435 | 0.079 | -0.1187 | No |
| 22 | SDC3 | na | 10638 | 0.076 | -0.1208 | No |
| 23 | PVR | na | 11152 | 0.068 | -0.1323 | No |
| 24 | HLA-DOA | na | 11639 | 0.061 | -0.1434 | No |
| 25 | CLDN14 | na | 11888 | 0.057 | -0.1478 | No |
| 26 | CDH1 | na | 11891 | 0.057 | -0.1450 | No |
| 27 | MPZL1 | na | 12042 | 0.055 | -0.1467 | No |
| 28 | VCAN | na | 12408 | 0.050 | -0.1548 | No |
| 29 | CLDN4 | na | 12650 | 0.047 | -0.1594 | No |
| 30 | ALCAM | na | 12716 | 0.046 | -0.1591 | No |
| 31 | CLDN5 | na | 13076 | 0.041 | -0.1674 | No |
| 32 | F11R | na | 13197 | 0.040 | -0.1690 | No |
| 33 | ICAM1 | na | 13556 | 0.036 | -0.1776 | No |
| 34 | CLDN15 | na | 13558 | 0.035 | -0.1758 | No |
| 35 | NEGR1 | na | 13619 | 0.035 | -0.1759 | No |
| 36 | HLA-DMB | na | 13802 | 0.033 | -0.1795 | No |
| 37 | HLA-F | na | 13970 | 0.031 | -0.1828 | No |
| 38 | HLA-DMA | na | 14233 | 0.028 | -0.1890 | No |
| 39 | ICOSLG | na | 14469 | 0.025 | -0.1946 | No |
| 40 | CD276 | na | 14800 | 0.021 | -0.2031 | No |
| 41 | SELP | na | 15110 | 0.018 | -0.2111 | No |
| 42 | SDC4 | na | 15221 | 0.017 | -0.2135 | No |
| 43 | CDH5 | na | 15250 | 0.017 | -0.2135 | No |
| 44 | NLGN2 | na | 15319 | 0.016 | -0.2147 | No |
| 45 | HLA-E | na | 15330 | 0.016 | -0.2142 | No |
| 46 | HLA-A | na | 15413 | 0.015 | -0.2158 | No |
| 47 | CLDN8 | na | 15477 | 0.014 | -0.2170 | No |
| 48 | SELE | na | 15676 | 0.012 | -0.2221 | No |
| 49 | SDC1 | na | 16391 | 0.005 | -0.2425 | No |
| 50 | ICOS | na | 16429 | 0.004 | -0.2434 | No |
| 51 | CNTNAP1 | na | 16723 | 0.001 | -0.2518 | No |
| 52 | HLA-B | na | 17543 | -0.007 | -0.2751 | No |
| 53 | MAG | na | 17785 | -0.009 | -0.2816 | No |
| 54 | HLA-G | na | 18423 | -0.015 | -0.2993 | No |
| 55 | NFASC | na | 18473 | -0.015 | -0.3000 | No |
| 56 | ITGA6 | na | 18594 | -0.016 | -0.3026 | No |
| 57 | HLA-C | na | 18927 | -0.019 | -0.3113 | No |
| 58 | ITGAL | na | 19153 | -0.022 | -0.3167 | No |
| 59 | HLA-DRB5 | na | 19471 | -0.025 | -0.3247 | No |
| 60 | CD6 | na | 19488 | -0.025 | -0.3239 | No |
| 61 | NLGN4X | na | 20092 | -0.031 | -0.3398 | No |
| 62 | PECAM1 | na | 20351 | -0.033 | -0.3456 | No |
| 63 | CLDN19 | na | 20448 | -0.034 | -0.3467 | No |
| 64 | CADM1 | na | 20660 | -0.036 | -0.3510 | No |
| 65 | CLDN1 | na | 21068 | -0.041 | -0.3608 | No |
| 66 | NCAM2 | na | 21398 | -0.044 | -0.3681 | No |
| 67 | ITGB1 | na | 21444 | -0.045 | -0.3672 | No |
| 68 | NEO1 | na | 22177 | -0.053 | -0.3858 | No |
| 69 | CLDN9 | na | 22183 | -0.053 | -0.3834 | No |
| 70 | GLG1 | na | 22246 | -0.053 | -0.3825 | No |
| 71 | CD2 | na | 23036 | -0.062 | -0.4023 | No |
| 72 | ICAM3 | na | 23345 | -0.066 | -0.4080 | No |
| 73 | HLA-DRB1 | na | 23407 | -0.067 | -0.4064 | No |
| 74 | CD40 | na | 23601 | -0.070 | -0.4086 | No |
| 75 | HLA-DRB3 | na | 23619 | -0.070 | -0.4056 | No |
| 76 | CLDN23 | na | 24003 | -0.075 | -0.4130 | No |
| 77 | ESAM | na | 24505 | -0.083 | -0.4234 | No |
| 78 | ITGAV | na | 24631 | -0.085 | -0.4229 | No |
| 79 | NLGN1 | na | 24981 | -0.090 | -0.4286 | No |
| 80 | PTPRC | na | 25329 | -0.096 | -0.4339 | No |
| 81 | CLDN3 | na | 25436 | -0.097 | -0.4322 | No |
| 82 | CD58 | na | 25548 | -0.099 | -0.4305 | No |
| 83 | ICAM2 | na | 25767 | -0.103 | -0.4318 | No |
| 84 | NRXN2 | na | 26320 | -0.113 | -0.4422 | No |
| 85 | SIGLEC1 | na | 26554 | -0.117 | -0.4432 | No |
| 86 | HLA-DOB | na | 26792 | -0.122 | -0.4441 | No |
| 87 | ITGB2 | na | 27251 | -0.131 | -0.4509 | No |
| 88 | CLDN18 | na | 27845 | -0.145 | -0.4609 | No |
| 89 | CTLA4 | na | 28101 | -0.151 | -0.4609 | No |
| 90 | ITGA4 | na | 28386 | -0.158 | -0.4613 | No |
| 91 | CD274 | na | 28392 | -0.158 | -0.4537 | No |
| 92 | CLDN11 | na | 29057 | -0.178 | -0.4641 | Yes |
| 93 | CNTN1 | na | 29247 | -0.183 | -0.4606 | Yes |
| 94 | CD8A | na | 29570 | -0.193 | -0.4604 | Yes |
| 95 | PTPRM | na | 29591 | -0.194 | -0.4515 | Yes |
| 96 | L1CAM | na | 29919 | -0.204 | -0.4510 | Yes |
| 97 | CD40LG | na | 30110 | -0.210 | -0.4462 | Yes |
| 98 | HLA-DRB4 | na | 30111 | -0.210 | -0.4359 | Yes |
| 99 | CNTNAP2 | na | 30599 | -0.227 | -0.4388 | Yes |
| 100 | HLA-DPA1 | na | 30869 | -0.237 | -0.4350 | Yes |
| 101 | ITGB8 | na | 30877 | -0.237 | -0.4236 | Yes |
| 102 | JAM2 | na | 30879 | -0.237 | -0.4120 | Yes |
| 103 | CD8B | na | 30954 | -0.240 | -0.4023 | Yes |
| 104 | CD22 | na | 31102 | -0.246 | -0.3945 | Yes |
| 105 | HLA-DQB1 | na | 31211 | -0.250 | -0.3853 | Yes |
| 106 | HLA-DRA | na | 31261 | -0.252 | -0.3744 | Yes |
| 107 | CDH15 | na | 31840 | -0.282 | -0.3773 | Yes |
| 108 | NRCAM | na | 32073 | -0.296 | -0.3694 | Yes |
| 109 | HLA-DQA1 | na | 32266 | -0.307 | -0.3599 | Yes |
| 110 | CLDN17 | na | 32373 | -0.315 | -0.3475 | Yes |
| 111 | SPN | na | 32388 | -0.316 | -0.3324 | Yes |
| 112 | CLDN10 | na | 32614 | -0.332 | -0.3226 | Yes |
| 113 | CD34 | na | 32709 | -0.338 | -0.3087 | Yes |
| 114 | CLDN20 | na | 32838 | -0.348 | -0.2953 | Yes |
| 115 | CD226 | na | 33045 | -0.363 | -0.2834 | Yes |
| 116 | NRXN3 | na | 33050 | -0.364 | -0.2657 | Yes |
| 117 | NLGN3 | na | 33096 | -0.368 | -0.2489 | Yes |
| 118 | NCAM1 | na | 33117 | -0.369 | -0.2314 | Yes |
| 119 | CDH2 | na | 33306 | -0.386 | -0.2179 | Yes |
| 120 | HLA-DQA2 | na | 33332 | -0.388 | -0.1995 | Yes |
| 121 | HLA-DPB1 | na | 33623 | -0.423 | -0.1872 | Yes |
| 122 | CLDN2 | na | 33643 | -0.424 | -0.1669 | Yes |
| 123 | ITGA8 | na | 33657 | -0.426 | -0.1464 | Yes |
| 124 | CDH4 | na | 33900 | -0.463 | -0.1306 | Yes |
| 125 | JAM3 | na | 33927 | -0.468 | -0.1085 | Yes |
| 126 | VCAM1 | na | 34132 | -0.510 | -0.0893 | Yes |
| 127 | CADM3 | na | 34212 | -0.530 | -0.0656 | Yes |
| 128 | SELL | na | 34237 | -0.538 | -0.0399 | Yes |
| 129 | MADCAM1 | na | 34279 | -0.550 | -0.0141 | Yes |
| 130 | NRXN1 | na | 34281 | -0.551 | 0.0129 | Yes |
Table: GSEA details [plain text format]

  

Fig 2: KEGG\_CELL\_ADHESION\_MOLECULES\_CAMS      
 Blue-Pink O' Gram in the Space of the Analyzed GeneSet

  

Fig 3: KEGG\_CELL\_ADHESION\_MOLECULES\_CAMS: Random ES distribution      
 Gene set null distribution of ES for **KEGG\_CELL\_ADHESION\_MOLECULES\_CAMS**
